# Passive dispersal potential of medaka eggs by attaching to waterbirds

**DOI:** 10.1007/s00114-024-01935-3

**Published:** 2024-10-01

**Authors:** Akifumi Yao, Miyuki Mashiko, Yukihiko Toquenaga

**Affiliations:** 1 College of Biological Sciences, School of Life and Environmental Sciences, University of Tsukuba, Tsukuba, Ibaraki 305-8572 Japan; 2 Present Address: Misaki Marine Biological Station, Graduate School of Science, The University of Tokyo, Misaki, Miura, Kanagawa 238-0225 Japan; 3 Animal Welfare and Wildlife Damage Management Group, Institute of Livestock and Grassland Science, National Agriculture and Food Research Organization, Tsukuba, Ibaraki 305-8666 Japan; 4 Institute of Life and Environmental Sciences, University of Tsukuba, Tsukuba, Ibaraki 305-8572 Japan

**Keywords:** Colonization, Epizoochory, Freshwater fish, Grey Heron, Population dynamics, Wetland

## Abstract

Colonization of new habitats is a key event in forming current distributions in organisms. It has been speculated that freshwater fish eggs can be dispersed passively by attaching to or egestion from waterbirds that arrive in wetland habitats. Recent research showed that some freshwater fish eggs could be excreted alive from birds and then successfully hatch, but scientific evidence of bird-mediated fish dispersal is still limited to endozoochory (internal transport through a bird’s digestive tract). Here, we experimentally suggest the dispersal potential in another way or epizoochory (external dispersal by attaching to waterbirds), using medaka *Oryzias latipes*, which spawns on aquatic plants. Our field experiment showed that waterbirds could carry artificial aquatic plants among waterbodies. Medaka eggs attached to aquatic plants could survive in the air for up to 18 h with a median lethal period of 16.3 h. Those two findings raise the possibility of the epizoochory of medaka in nature.

**Supplementary Information:**

The online version contains supplementary material available at 10.1007/s00114-024-01935-3.

## Introduction

Dispersal over geographic barriers plays an essential role in colonization, gene flow, metapopulation dynamics, and invasion (Bowler and Benton 2005). Since dry lands strictly separate freshwater habitats, as expressed by the phrase “islands of water in a sea of dry land” (Faulks et al. 2010), dispersal among freshwater waterbodies by themselves is challenging for aquatic organisms such as freshwater fishes. Although connecting waterbodies by geographic events (i.e., stream captures, sea-level changes) and disasters (floods) also cause such dispersal (Thomaz et al. 2007; Dias et al. 2014; Willett et al. 2014), it has long been speculated worldwide that freshwater fish eggs are dispersed by attaching to (epizoochory or ectozoochory) or egestion from (endozoochory) waterbirds (Hirsch et al. 2018). It is well documented that waterbirds disperse aquatic plants, zooplankton, and various invertebrates both by epizoochory and endozoochory (Coughlan et al. 2017a; Green et al. 2023). However, there is only three reported cases of empirical evidence of endozoochory in freshwater fishes and no scientific evidence of epizoochory (Hirsch et al. 2018; Silva et al. 2019; Lovas-Kiss et al. 2020, 2024; Green et al. 2023).

In this study, we focused on the southern medaka *Oryzias latipes* (hereafter medaka) as a case study. Medaka is a small freshwater fish living in shallow waterbodies in Japan except Hokkaido Island (Senou 2013). This species lays eggs on various substrates, such as submerged aquatic plants (Iwata et al. 2015). The picture book *Flying Medaka in the Sky* (“*Soratobu medaka*” in Japanese) reported that shoals of medaka were found in a shallow concrete pool that was used to wash trucks. The writer proposed waterbirds such as night herons (*Nycticorax nycticorax*) carried medaka eggs from a nearby stream because he thought that only birds could reach both the concrete pool and the creek, and he was convinced that such waterbirds carried eggs attached to aquatic plants tangled on their feet (Nakamura 1999).

Here, we experimentally investigate this scenario and suggest that medaka eggs potentially travel among isolated waterbodies passively by attaching to waterbirds. Epizoochory can be achieved through the following five steps: (1) encounter to the vector, (2) attachment to the vector, (3) survival on the vector during dispersal, (4) detachment at a new habitat, and (5) colonization at the new habitat (Coughlan et al. 2017a). Our experiments mainly deal with steps 1 and 2 from the field experiment and step 3 from the egg dehydration experiment.

## Materials and methods

### Field experiment

**Fig. 1:**
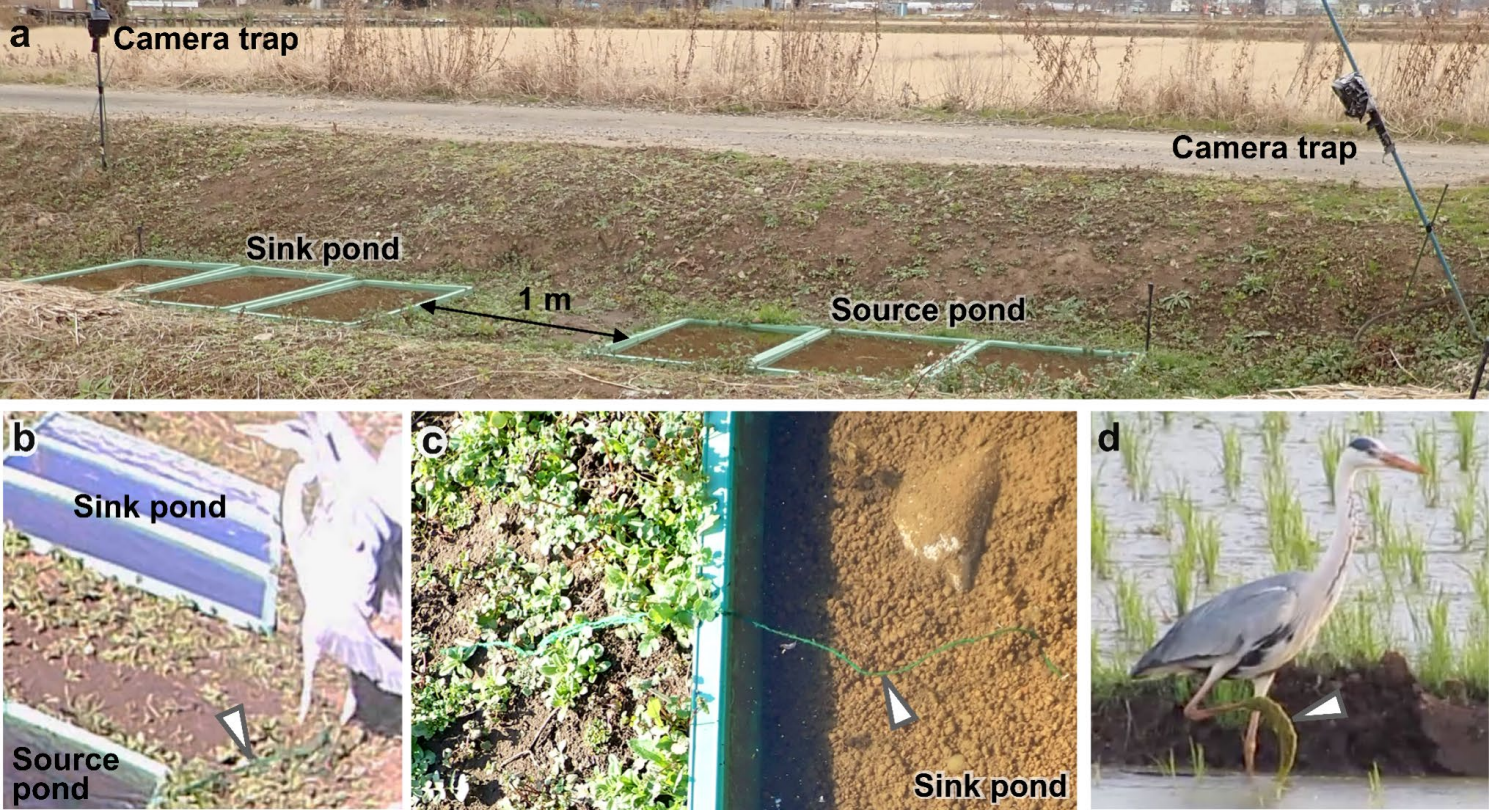
The field experiment suggests waterbirds could carry aquatic plants that serve medaka’s spawning substrates. **a** Field experimental site. **b** A grey heron hooked artificial aquatic plants on its foot and flew away. Arrowhead shows artificial aquatic plants (from Online Resource 4). **c** An artificial aquatic plant was found at the sink pond. Arrowhead shows the artificial aquatic plant. **d** A grey heron carried clumps of algae on its foot and walked in paddy fields. Arrowhead shows algae on heron foot (from Online Resource 6)

To examine whether waterbirds carry submerged aquatic plants that serve as medaka’s spawning substrates, field experiments were conducted in 2019. We constructed two experimental ponds about 1 m × 2 m with a 1-m distance (Fig. 1a). To prevent genetic disturbance of local populations, we used artificial aquatic plants to substitute for submerged aquatic plants and did not attach medaka’s egg. To attract waterbirds such as herons and egrets, small baitfish were added to these ponds (about 10 individuals/plastic container). Thirty-six artificial plants were placed in one of the ponds (source pond), and we observed whether the waterbirds dispersed them to the other pond (sink pond) by motion capture camera traps.

### Egg dehydration experiment

To evaluate the survivability of medaka eggs in the air, clutches of eggs were attached to 5-cm pieces of aquatic plants (*Egeria densa*) or placed on slide glasses. Then, they were exposed to the air for 0–24 h with 75% relative humidity and 25 °C, similar to the weather conditions of medaka’s reproductive season. After the treatment, eggs were reared, and hatching rates were calculated. Detailed materials and methods are described in the Online Resource 1.

## Results

In the field experiment, the camera trap recorded that a grey heron (*Ardea cinerea*) hooked the artificial plants on its foot and flew away from the sink pond (December 5, 2019, Fig. 1b, Online Resource 2, 3, 4). The artificial aquatic plants were observed around experimental ponds that were located up to 6 m from the source pond (observed and measured on December 8, 2019). In addition, an artificial aquatic plant was found in the sink pond on another day (Fig. 1c, observed on December 14, 2019). Although the detailed traveling process of the plant was not recorded, one heron walked from the source pond to sink pond before this observation (December 11, 2019, Online Resource 5). Also, there was no heavy rainfall, and the flooding did not cause the two ponds to connect at this time. Furthermore, we happened to observe a grey heron walking between paddy fields with a clump of algae on its foot near experimental ponds on May 23, 2019 (Fig. 1d, Online Resource 6).

**Fig. 2:**
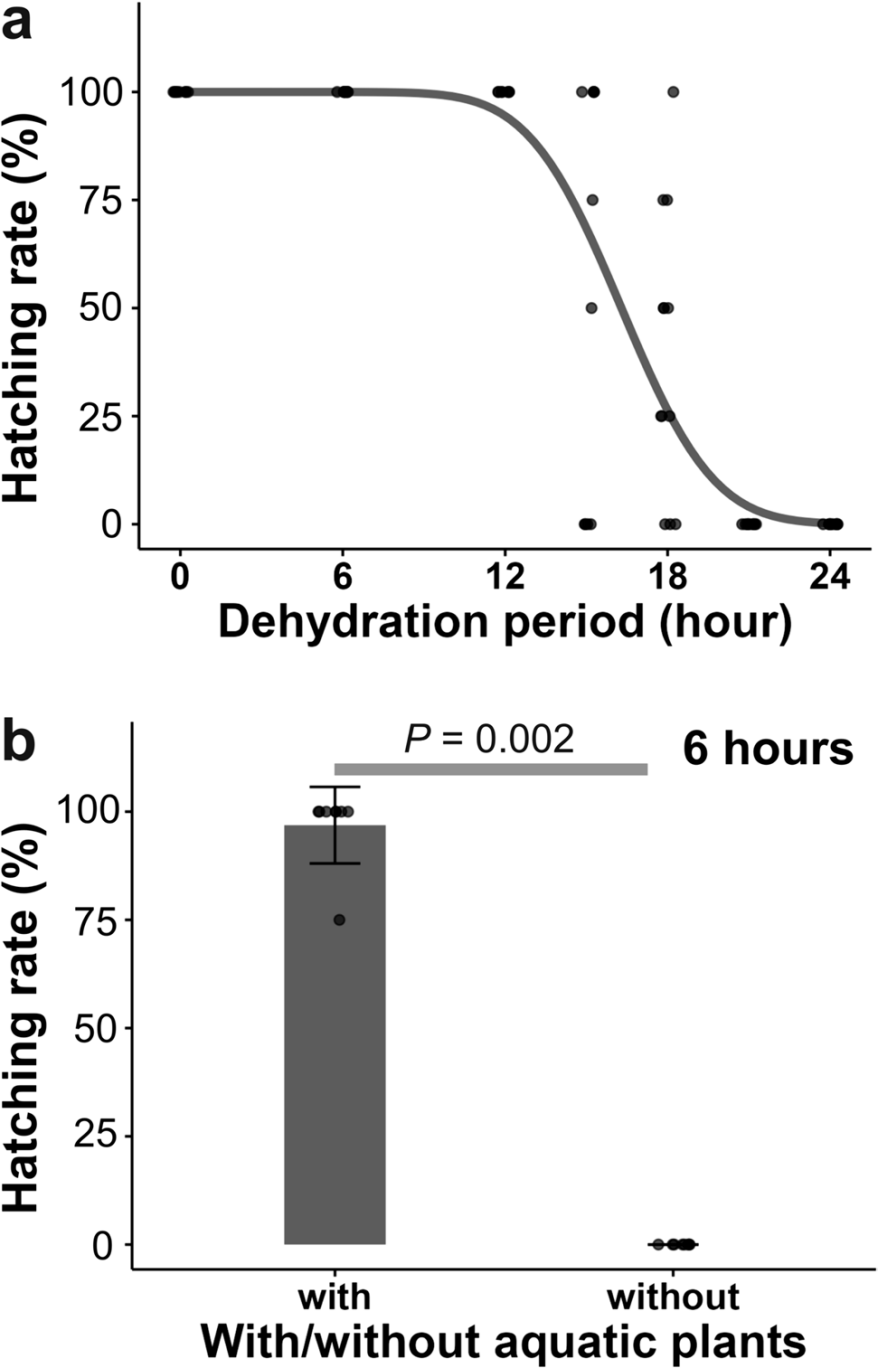
Hatchability of medaka eggs after exposure to the air. **a** Changes of hatching rates of eggs attached to aquatic plants with different air exposure periods. **b** Hatching rates of eggs after 6-h air exposure with and without aquatic plants

Next, we examined whether medaka eggs attached to aquatic plants could survive in the air. When eggs were attached to aquatic plants, they could survive in the air for up to 18 h and then hatch. Estimated median lethal period was 16.3 ± 0.3 h (Fig. 2a, *N*= 7–12 replicates with four eggs in each replicate, probit regression, *Z* = 0.048, *P* < 0.001). In contrast, all eggs died when they were in the air for 6 h without being attached to aquatic plants (Fig. 2b, *N* = 8 replicates with four eggs in each replicate, hatching rate of eggs with aquatic plants: 96.9%, that of without aquatic plants: 0%, Wilcoxon signed-rank test, *Z* = 3.051, *P* = 0.002).

## Discussion

The present findings suggest that medaka eggs attached to aquatic plants can potentially be dispersed by waterbirds, and the scenario proposed by the picture book (Nakamura 1999) may be true. Our field experiment and observation show that aquatic plants can be dispersed passively by attaching to waterbirds (Fig. 1). These results also imply medaka eggs can potentially be carried between waterbodies if attached to such aquatic plants. Previous studies reported that seeds of various aquatic plants and green plant materials of small floating aquatic plants could be dispersed by bird-mediated epizoochory (Coughlan et al. 2017a, b). However, there have been no reports of epizoochory on green plant materials of submerged aquatic plants that serve as spawning substrates for medaka, although their dehydration tolerance has been investigated to evaluate their potential for epizoochory, and it has been reported that waterbirds carry seeds of such aquatic plants among continents by endozoochory (Viana et al. 2013; Coughlan et al. 2018). Our observations suggest that the green plant materials of submerged macrophytes can also be dispersed by epizoochory. In this study, the dispersal of aquatic plants was observed in December, which is not the reproductive season of medaka. However, herons and egrets fly to shallow waterbodies, which are suitable habitats for medaka, for foraging throughout the year, including medaka’s reproductive season from April to September (Iwamatsu 2004). Therefore, dispersal of aquatic plants can occur during the reproductive season of medaka.

The egg dehydration experiment shows that medaka eggs that are attached to aquatic plants can survive in the air for up to 18 h, but all eggs die when they are in the air for 6 h without aquatic plants (Fig. 2). These results suggest that their eggs do not have strong desiccation tolerance but could survive out of the water for a certain period by attaching to moist aquatic plants. Temperature and humidity can affect survivability in the air, which may need investigations in a wide range of conditions. Similar to our results, Oulton et al. (2013) also showed that moist substrates enhance the hatching success of crimson spotted rainbowfish (*Melanotaenia duboulayi*) egg after exposure to the air.

This study, together with previous studies, suggests that waterbirds can carry the eggs of various freshwater fishes. Previous research showed that killifish eggs with strong chorion and four freshwater fishes’ eggs (Cypriniformes) that have soft chorion could be dispersed by endozoochory (Silva et al. 2019; Lovas-Kiss et al. 2020, 2024). Our study suggests the potential that medaka eggs spawned on aquatic plants can be dispersed by attaching to waterbirds. Surveys of artificial ponds in French suggested that waterbirds may disperse European perch’s eggs (*Perca fluviatilis*) (Garcia et al. 2023). Taken together, these findings show that various freshwater fishes spawned in shallow water can be dispersed both by endozoochory and epizoochory, suggesting that birds potentially disperse a wide range of freshwater fishes.

Overall, our field and laboratory experiments suggest the epizoochory potential of a freshwater fish, medaka, by waterbirds. In our field experiment, living aquatic plants and fish eggs were replaced with artificial aquatic plants to avoid genetic disturbance to the local plant and animal populations around the experimental site. Future experiments in controlled closed environments, such as zoos, using living aquatic plants and fish eggs, will overcome the restriction of field trial and provide more direct evidence of epizoochory in medaka and freshwater fishes. Also, high-resolution population genetic studies will help to elucidate how waterbirds contribute to assemblies of fish populations in freshwater habitats.

## Supplementary Information

Below is the link to the electronic supplementary material.

Supplementary file1 (DOCX 28 KB)

Supplementary file2 (DOCX 22 KB)

Supplementary file3 (MOV 9600 KB)

Supplementary file4 (MOV 3730 KB)

Supplementary file5 (MOV 23020 KB)

Supplementary file6 (MOV 9855 KB)

## Data Availability

Data and code are deposited in Zenodo Repository (10.5281/zenodo.10784845) and GitHub (https://github.com/yaoakifumi/Medaka-embryo-desication-tolerance), respectively.
