# Supplementary material for: Passive dispersal potential of medaka eggs by attaching to waterbirds
Source: Naturwissenschaften. 2024 Oct 1;111(5):53. doi: 10.1007/s00114-024-01935-3 (PMC11445353; doi:10.1007/s00114-024-01935-3)
Supplement: Supplementary file 1 — Supplementary file1 (DOCX 28 KB) [file 114_2024_1935_MOESM1_ESM.docx]

**SUPPLEMENTAL INFORMATION (ESM1)**

The Science of Nature, short communication

Passive dispersal potential of medaka eggs by attaching to waterbirds

Akifumi Yao, Miyuki Mashiko, and Yukihiko Toquenaga.

Corresponding author: Akifumi Yao, yao@mmbs.s.u-tokyo.ac.jp

Misaki Marine Biological Station, Graduate School of Science, The University of Tokyo, Misaki, Miura, Kanagawa, 238-0225 Japan

**Supplemental Materials and Methods**

**Ethical statement**

Experiments in this study followed the ARRIVE guideline, the animal experiment use guidelines of University of Tsukuba (https://www.md.tsukuba.ac.jp/LabAnimalResCNT/kitei/doubutsukitei.pdf) and that of The University of Tokyo (https://www.u-tokyo.ac.jp/gen01/reiki_int/reiki_honbun/au07404001.html).

**Field Experiment**

For investigating whether waterbirds could carry aquatic plants that serve as medaka’s spawning substrates, we constructed two experimental ponds (1 m × 2 m, 1 m distance between ponds) by burying plastic containers (91 cm × 62 cm × 20 cm; three containers in one pond) in a shallow irrigation ditch among paddy fields in Hojo, Tsukuba, Ibaraki, Japan. Akadama sand was laid down on the bottom of the ponds. The water depth of these ponds was about 10 cm. To prevent genetic disturbance of local populations by escaping living aquatic plants and medaka eggs that were collected from outside the experimental site, we substituted artificial aquatic plants for submerged aquatic plants and did not attach medaka egg. Thirty-six “strap-shaped” artificial aquatic plants (length: about 30 cm, made from polypropylene, Suisaku Co., Ltd., Japan) were placed in the “source pond.” Herons and egrets constantly visited paddy fields in the Hojo area for foraging. Since herons and egrets are large waterbirds compared with other birds that came flying in paddy fields, such as spot-billed duck (*Anas zonorhyncha*) and Japanese wagtail (*Motacilla grandis*), and it was thus expected that such large shorebirds would relatively easily carry aquatic plants, we focused on them. It is known that herons and egrets eat fish, frogs, crustaceans, insects, and other small animals (Ogasawara et al. 1982; Tojo 1996). Therefore, to attract herons and egrets, small baitfish (topmouth gudgeon, *Pseudorasbora parva*, continental rosy bitterling, *Rhodeus ocellatus ocellatus*, and japanese weatherfish, *Misgurnus sp*., collected in Tsukuba city) were added to these ponds, and replaced in response to their absence (about 10 individuals/plastic container). Arrivals of animals and their behaviors around ponds were recorded by two motion capture camera traps (CMS-SC03GY，SANWA SUPPLY Inc., Okayama, Japan). The recording parameter of the cameras was to record 60 seconds per each bird/animal arrival (detected by infrared sensors) for the whole day during the experimental period.

**Egg dehydration treatment**

To examine whether medaka eggs attached to aquatic plants could survive in the air during dispersal by birds, egg dehydration experiments were conducted. Parental medaka were collected using a hand-net at the pond in the Yata River system, Tsukuba, Ibaraki, Japan in October 2023. Each pair of medaka was reared for collecting eggs in a separate tank (32 cm× 19 cm× 24 cm) with filtering and aeration at a water temperature of 26°C; light period: dark period (L: D) =14h:10h. Egg clutches were collected every morning and stored in freshwater supplemented with 0.1% methylene blue (rearing water) at 25°C, L:D=12h:12h in a six-well dish in an incubator (MTI-201, Tokyo Rikakikai Co., Ltd., Japan). Four eggs from each clutch were kept in rearing water as a control. If the control eggs did not hatch, we removed the data based on this clutch. Since control eggs did not hatch or rearing water dried up accidentally, three data points were removed from the dataset. Clutches one day after spawning were used for the experiment. Since medaka eggs have long adhesive filaments (Iwamatsu 2004), four eggs were attached to a 5-cm piece of Anacharis *Egeria densa* by tangling their adhesive filaments to plant materials. Then, eggs were exposed to the air with 75% relative humidity and 25°C in an incubator (MIR-154, SANYO Electric Co. Ltd., Japan). Such environmental conditions were similar to the average humidity and temperature recorded in Tsukuba by the Japan Meteorological Agency in July, which was medaka’s reproductive season. Humidity was maintained using a saturated saline solution in the incubator. Temperature and humidity were checked using Thermo-hygrometer (IBS-TH1-mini, Inkbird, China). Seven exposure times were applied (0, 6, 12, 15, 18, 21, and 24 hours). After treatment, eggs were kept in rearing water until they hatch. Usually, medaka eggs hatch after about 10 days at 26°C (Iwamatsu 2004). Each treatment was performed with seven to 12 biological replicates. In addition, four eggs were exposed to the air with (attached to 5 cm of Anacharis) and without (placed on the sliding glass) aquatic plants to evaluate the importance of moisture retention by attachment to aquatic plants for egg survival (N = 8 biological replicates).

**Statistical analysis**

Statistical analysis was performed using R 4.3.1 (R Core Team 2023). We examined the association of hatching rate and the period in the air using probit regression model following the previous study (Banha and Anasta 2012). Median lethal time (the time of 50% mortality or LD_50_) of exposed air was calculated based on hatching rate. Also, hatching rates with and without aquatic plants were compared using Wilcoxon signed-rank test.

**References**

Banha F, Anasta PM (2012) Waterbird-mediated passive dispersal of river shrimp *Athyaephyra desmaresti*. Hydrobiologia 694:197–204. https://doi.org/10.1007/s10750-012-1160-7

Iwamatsu T (2004) Stages of normal development in the medaka *Oryzias latipes*. Mech Dev 121:605–618. https://doi.org/10.1016/j.mod.2004.03.012

Ogasawara K, Abe K, Naito T (1982) Ecological study of grey heron in oga peninsula, akita prefecture. J Yamashina Inst Ornithol 14:232–245. https://doi.org/10.3312/jyio1952.14.232

R Core Team (2023) R: A language and environment for statistical computing. R foundation for statistical computing, Vienna, Austria. https://www.R-project.org/.

Tojo H (1996) Habitat selection, foraging behaviour and prey of five heron species in Japan. Japanese J Ornithol 45:141–158. https://doi.org/10.3838/jjo.45.141
