# Supplementary material for: Passive dispersal potential of medaka eggs by attaching to waterbirds
Source: Naturwissenschaften. 2024 Oct 1;111(5):53. doi: 10.1007/s00114-024-01935-3 (PMC11445353; doi:10.1007/s00114-024-01935-3)
Supplement: Supplementary file 2 — Supplementary file2 (DOCX 22 KB) [file 114_2024_1935_MOESM2_ESM.docx]

**SUPPLEMENTAL INFORMATION (ESM2)**

The Science of Nature, short communication

Passive dispersal potential of medaka eggs by attaching to waterbirds

Akifumi Yao, Miyuki Mashiko, and Yukihiko Toquenaga.

Corresponding author: Akifumi Yao, yao@mmbs.s.u-tokyo.ac.jp

Misaki Marine Biological Station, Graduate School of Science, The University of Tokyo, Misaki, Miura, Kanagawa, 238-0225 Japan

**Captions of supplemental videos**

**Online Resource 3 (Video S1).**

A grey heron hooked artificial aquatic plants on its leg and was walking in the sink pond (experimental pond). This video was taken by a motion capture camera trap. Recorded on Dec. 5, 2019, in Hojo, Tsukuba, Ibaraki, Japan.

Videographer: Akifumi Yao

**Online Resource 4 (Video S2).**

A grey heron hooked artificial aquatic plants on its leg and flew away from the sink pond (experimental pond). This video was taken by a motion capture camera trap. Recorded on Dec. 5, 2019, in Hojo, Tsukuba, Ibaraki, Japan.

Videographer: Akifumi Yao

**Online Resource 5 (Video S3).**

A grey heron walked from the source pond to the sink pond (experimental ponds). This video was taken by a motion capture camera trap. Recorded on Dec. 11, 2019, in Hojo, Tsukuba, Ibaraki, Japan.

Videographer: Akifumi Yao

**Online Resource 6 (Video S4).**

A grey heron walked between paddy fields tangled clump of algae on its leg. Recorded on May 23, 2019, in Hojo, Tsukuba, Ibaraki, Japan.

Videographer: Miyuki Mashiko
